# Extensive Pustular Tinea Incognita During Late Pregnancy: A Case Report

**DOI:** 10.7759/cureus.111273

**Published:** 2026-06-22

**Authors:** Beatriz F Vilela, MC Fialho, José M Neves

**Affiliations:** 1 Dermatology, Unidade Local de Saúde de São José, Lisbon, PRT

**Keywords:** dermatophytosis, pregnancy, pustular eruption, tinea corporis, tinea incognita

## Abstract

Tinea incognita is an atypical presentation of dermatophytosis, usually modified by prior corticosteroid exposure, which may obscure the clinical diagnosis and mimic inflammatory dermatoses. We report the case of a 27-year-old woman at 35 weeks of gestation who presented with a three-week history of painful inflammatory lesions involving the gluteal region and posterior thighs. The eruption initially presented as bilateral erythema of the inguinal folds and gluteal area and worsened rapidly after topical mometasone treatment. Physical examination revealed extensive, symmetrical, well-demarcated erythematous plaques with fine scaling and numerous peripheral pustules. Given the pustular morphology and pregnancy context, pustular psoriasis was initially considered. However, direct mycological examination was positive, and fungal culture subsequently identified *Trichophyton rubrum*, confirming the diagnosis of pustular tinea incognita. Treatment with oral itraconazole led to marked clinical improvement. This case highlights the importance of considering dermatophytosis in the differential diagnosis of pustular eruptions during pregnancy, particularly when lesions worsen after topical corticosteroid use. Early mycological testing can prevent diagnostic delay and inappropriate escalation of anti-inflammatory or immunosuppressive therapy.

## Introduction

Dermatophytosis is one of the most common superficial fungal infections worldwide and is caused by keratinophilic fungi of the genera *Trichophyton*, *Microsporum*, and *Epidermophyton*. Classical tinea corporis usually presents as annular erythematous plaques with peripheral scaling and central clearing. However, prior use of topical or systemic corticosteroids, calcineurin inhibitors, or other immunosuppressive treatments may alter the typical morphology of dermatophyte infection, leading to the entity known as tinea incognita [1,2].

Tinea incognita is a well-recognized diagnostic pitfall. By suppressing local inflammation, corticosteroids may reduce scaling and pruritus while allowing fungal proliferation and extension. As a result, lesions may become more extensive, less well demarcated, more inflammatory, pustular, eczematous, or psoriasiform, mimicking several inflammatory or infectious dermatoses [3-5]. Diagnostic delay is common, particularly when anti-inflammatory therapy is escalated before mycological confirmation.

Pregnancy adds further complexity to the diagnostic and therapeutic approach. Several inflammatory and pustular dermatoses may occur during gestation, including pustular psoriasis of pregnancy, candidal intertrigo, bacterial folliculitis, and drug-related eruptions. At the same time, treatment options must be selected carefully, considering both maternal benefit and fetal safety. For localized dermatophytosis during pregnancy, topical antifungals are generally preferred because of limited systemic absorption. However, extensive, severe, inflammatory, or refractory infections may require systemic treatment after careful maternal-fetal risk-benefit assessment, particularly in late pregnancy and in collaboration with obstetrics [6]. We report a case of pustular tinea incognita in late pregnancy, initially suspected to be pustular psoriasis, emphasizing the need to consider a dermatophyte infection in atypical inflammatory eruptions.

## Case presentation

A 27-year-old woman at 35 weeks of pregnancy, with no relevant personal or family dermatological history, presented with a three-week history of painful inflammatory lesions involving the gluteal region and proximal inner thighs. The lesions initially appeared as erythematous plaques. Before dermatology referral, topical mometasone cream had been used for three weeks. Despite treatment, the eruption progressed rapidly, with increasing erythema, pain, superficial scaling, and pustule formation.

**Figure 1:**
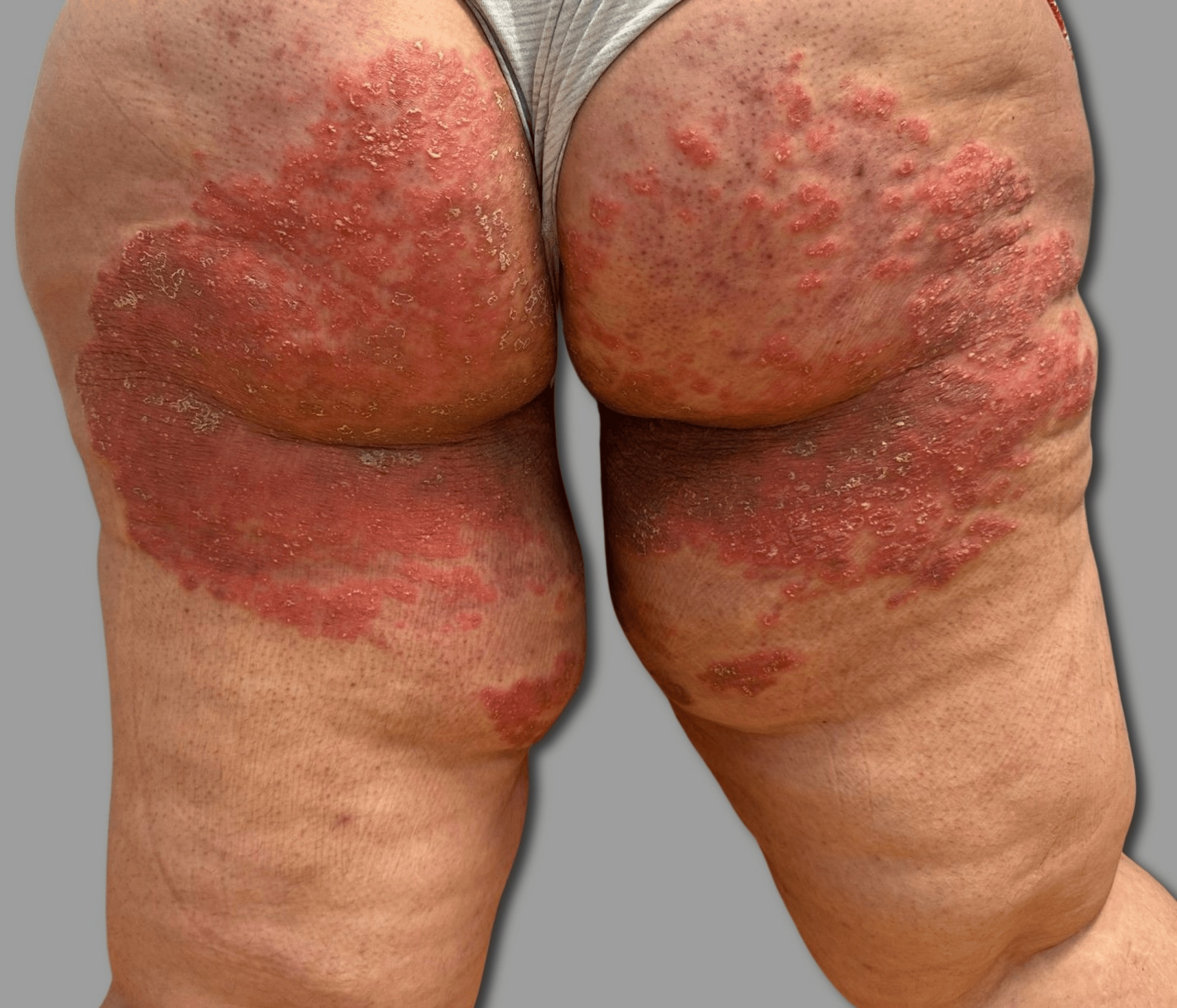
Clinical presentation of pustular tinea incognita Bilateral inflammatory erythematous plaques involving the gluteal region and proximal inner thighs, with superficial scaling and multiple peripheral pustules.

On dermatological examination, there were bilateral, relatively symmetrical erythematous plaques involving the gluteal region and extending to the inner thighs. The plaques showed superficial scaling and multiple pustules, predominantly located at the peripheral advancing border (Figure 1). There were no mucosal lesions, fever, malaise, or other systemic symptoms.

Given the gestational age and pustular morphology, pustular psoriasis of pregnancy was initially considered. However, the presence of peripheral scaling, centrifugal extension, and clinical worsening after topical corticosteroid use raised suspicion for a dermatophyte infection. Other differential diagnoses included candidal intertrigo, bacterial folliculitis, impetiginization, irritant or allergic contact dermatitis, inverse psoriasis, and acute generalized exanthematous pustulosis.

**Figure 2:**
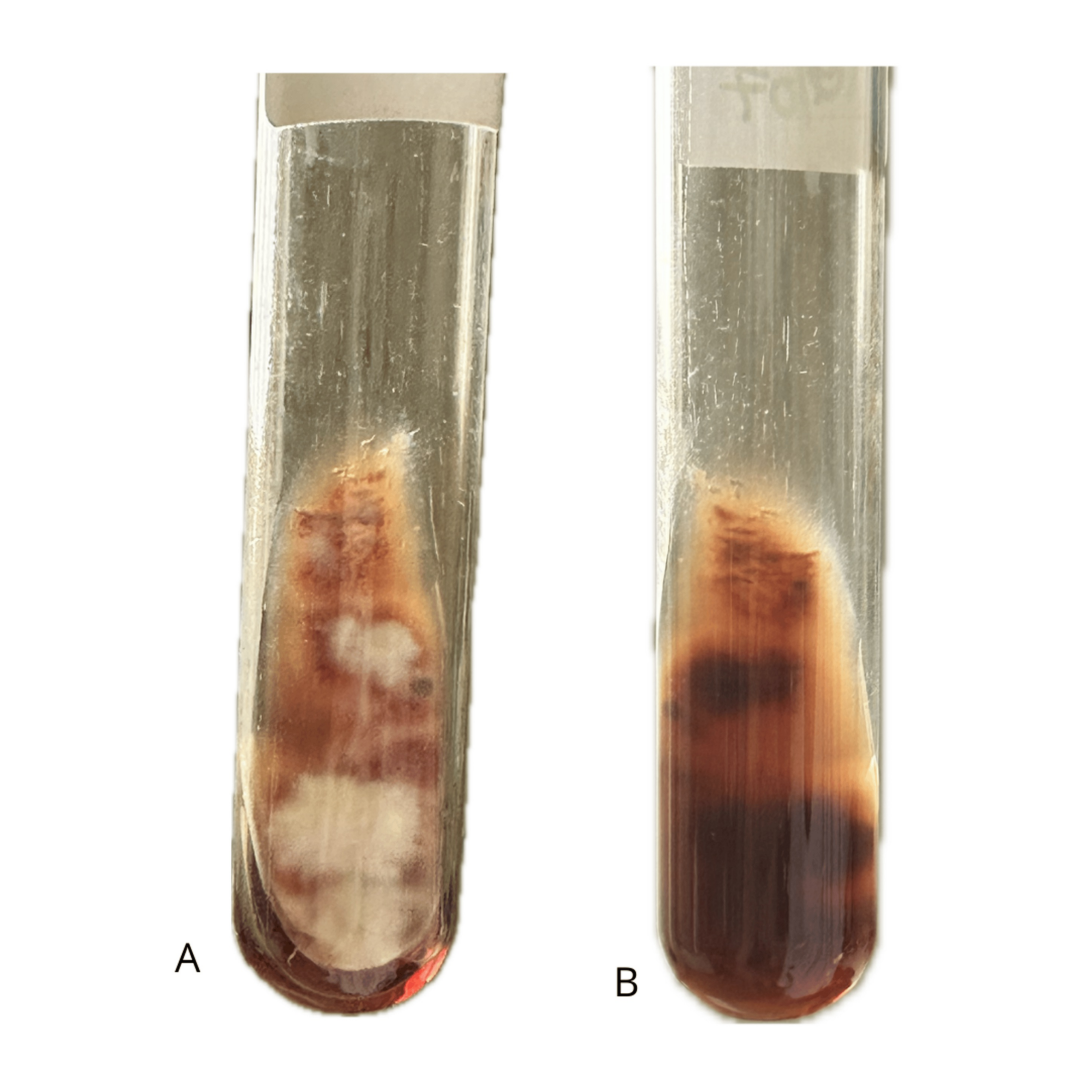
Fungal culture identifying Trichophyton rubrum Fungal culture of material obtained from skin scales and pustular contents, subsequently identified as *Trichophyton rubrum*. (A) Obverse view showing white cottony colony growth with reddish pigmentation. (B) Reverse view showing reddish pigment production.

Skin scale and pustular material were collected for direct mycological examination after application of 40% potassium hydroxide, which demonstrated fungal elements. Fungal culture (performed on Mycosel Agar® medium, Sabouraud dextrose agar, cycloheximide 0.4 g/L, and chloramphenicol 0.05 g/L) subsequently identified *Trichophyton rubrum*, confirming the diagnosis of pustular tinea incognita (Figure 2A-2B).

Topical corticosteroid therapy was discontinued. Considering the extent and painful inflammatory nature of the lesions, as well as the late gestational age, the case was discussed with the obstetrics team. After individualized maternal-fetal risk-benefit assessment, systemic antifungal therapy with itraconazole 200 mg/day was initiated for two weeks. At postpartum inpatient reassessment after four weeks, clinical improvement was documented, with reduction of erythema, pustulation, and pain. No maternal or fetal complications were reported during follow-up.

## Discussion

Tinea incognita refers to a dermatophyte infection with atypical clinical features resulting from prior exposure to corticosteroids or other immunosuppressive agents. The term reflects the masking of typical signs of tinea, which may lead to misdiagnosis and inappropriate treatment. Romano et al., in a 15-year survey of tinea incognita in Italy, emphasized the broad clinical spectrum of this entity and its frequent misdiagnosis as eczema, psoriasis, lupus erythematosus, or other inflammatory dermatoses [1]. Arenas et al. similarly highlighted that modified dermatophyte infections may present with reduced scaling, altered borders, and more extensive or inflammatory lesions [2].

Our case illustrates several diagnostic pitfalls. First, topical corticosteroid use prior to mycological confirmation likely altered the morphology and contributed to progression. Second, the pustular and intertriginous distribution in late pregnancy overlapped with pustular psoriasis of pregnancy, an important diagnosis to consider given its potential association with systemic symptoms and maternal-fetal complications. However, the absence of fever, malaise, mucosal involvement, or systemic deterioration made this diagnosis less likely. The presence of peripheral scale, centrifugal extension, and worsening after topical corticosteroid exposure are important clues to dermatophyte infection.

The differential diagnosis of pustular intertriginous plaques during pregnancy is broad. Candidal intertrigo may present with erythematous plaques and satellite pustules, particularly in occluded areas, but usually lacks the annular or centrifugal morphology of dermatophyte infection. Bacterial folliculitis or impetiginization may cause pustules and pain but typically does not present with scaly, advancing borders. Inverse psoriasis may affect intertriginous areas but is usually less scaly and does not show fungal elements on direct examination. Acute generalized exanthematous pustulosis is usually more widespread, often drug-related, and frequently associated with fever and neutrophilia. In this context, direct mycological examination and fungal culture were decisive.

Mycological confirmation remains essential in atypical inflammatory eruptions. Potassium hydroxide examination of scale or pustular material is rapid, inexpensive, and useful for immediate diagnostic orientation, while fungal culture allows species identification. In the present case, fungal culture showed features consistent with *Trichophyton rubrum*​​​​​, including white, cottony colony growth and reddish pigment production, followed by species identification. *Trichophyton rubrum* is among the most common dermatophytes implicated in tinea corporis and tinea incognita [1,4]. In pregnancy, confirmation is particularly relevant because it may prevent unnecessary escalation of corticosteroids or systemic anti-inflammatory therapy.

Treatment of dermatophytosis during pregnancy should be individualized based on the extent and severity of the disease, gestational age, available safety data, and maternal symptom burden. Topical antifungals, including topical azoles and allylamines, are generally preferred for localized disease because of limited systemic absorption. Systemic antifungal therapy is usually avoided when possible, particularly in early pregnancy, but may be considered in extensive, severe, inflammatory, or refractory disease after careful maternal-fetal risk-benefit assessment. In the present case, the patient was in late pregnancy and had extensive, painful, inflammatory lesions. The case was discussed with the obstetrics team, and the decision to proceed with systemic antifungal therapy was made jointly. In all cases of tinea incognita, withdrawal of inappropriate corticosteroid therapy is essential.

This case reinforces that pustular inflammation does not exclude dermatophyte infection. In pregnant patients with atypical inflammatory plaques, particularly when lesions worsen after corticosteroid exposure, tinea incognita should remain in the differential diagnosis, and mycological testing should be performed before further anti-inflammatory treatment.

## Conclusions

Tinea incognita is a frequent diagnostic pitfall and may closely mimic inflammatory dermatoses, including pustular psoriasis. Pregnancy further complicates the diagnostic approach because several pustular dermatoses may occur during gestation and treatment options require careful selection. In atypical, painful, pustular, or intertriginous plaques, especially those worsening after topical corticosteroid use, dermatophyte infection should be actively excluded. Direct mycological examination and fungal culture are simple yet crucial tools for avoiding diagnostic delay and inappropriate treatment.
